# Falls and Fear of Falling in Minor Stroke Patients With Vestibular Symptoms: A Longitudinal Observational Study

**DOI:** 10.1002/brb3.71509

**Published:** 2026-06-03

**Authors:** Yao Zhong, Jiaokun Jia, Jiashu Li, Ruixuan Jiang, Xingquan Zhao, Yi Ju

**Affiliations:** ^1^ Department of Neurology, Beijing Tiantan Hospital Capital Medical University Beijing China; ^2^ Clinical Center for Vertigo and Balance Disturbance Capital Medical University Beijing China

**Keywords:** falls, fear of falling, mild stroke, vestibular symptom

## Abstract

**Background and Purpose:**

Although stroke survivors are known to have an increased risk of chronic balance dysfunction and falls, prospective studies focusing on long‐term fall risk in mild stroke patients—especially those with acute vestibular symptoms—remain limited. This prospective study aimed to longitudinally characterize vestibular symptom resolution patterns within 1 year in minor stroke patients and identify predictors of the long‐term risk of falls or fear of falling (FoF).

**Methods:**

This prospective observational study recruited 242 patients with acute minor stroke presenting vestibular symptoms between September 2021 and December 2022. Vestibular symptom progression was systematically assessed at discharge, 3, 6, and 12 months poststroke. Functional outcomes were quantified using the modified Rankin Scale (mRS), while balance confidence and psychological status were evaluated with standardized instruments at each follow‐up. Longitudinal analysis tracked vestibular symptom evolution over 12 months. Multivariable logistic regression identified independent predictors of falls and FoF.

**Results:**

Among 242 minor stroke patients with acute vestibular symptoms, symptom persistence rates progressively declined from 56.20% at discharge to 30.99% at 3 months, 26.45% at 6 months, and 16.12% at 1‐year follow‐up. Multivariable regression revealed that a total small vessel disease (SVD) score ≥ 2 (odds ratios [OR] = 5.106, 95% confidence intervals [CI]: 1.747–14.921, *p* = 0.003) independently predicted 1‐year fall risk. Notably, both persistent vestibular symptomsnd the presence of anxiety/depression poststroke were significantly associated with increased risks of falls and FoF.

**Conclusion:**

Vestibular symptoms resolution in minor stroke follows a biphasic trajectory, with rapid early improvement and slower late recovery. Importantly, temporal lobe involvement, SVD score ≥ 2, persistent vestibular symptoms, and anxiety/depression are independent predictors of long‐term fall risk. The findings highlight the clinical value of early SVD marker identification and regular monitoring of both vestibular function and emotional status.

## Graphical abstract

This prospective study reveals that persistent vestibular symptoms, cerebral small vessel disease, and anxiety/depression predict long‐term fall risk in minor stroke patients. Early identification and intervention within the first 3 months poststroke may help mitigate balance impairment and improve outcomes.

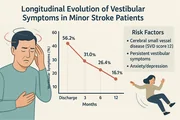

## 1 Introduction

With the advancement of medical conditions, the incidence of minor stroke has gradually increased (Perry et al. 2022). Approximately 3.7%–12.5% (Jahn et al. 2020; Navi et al. 2012; Park et al. 2023) of stroke patients experience vestibular symptoms such as dizziness or vertigo at onset. Although the symptoms of minor stroke patients are mild, and the risk of recurrence within 90 days has decreased from 10% to 1% (Amarenco et al. 2016; Rothwell et al. 2007), minor stroke patients with vestibular symptoms may face a significant risk of falls or fear of falling (FoF) during rehabilitation due to impaired vestibular function and decreased postural control (Roelofs et al. 2023; Xie et al. 2022).

Symptomatic dizziness (including problems with dizziness, balance, falls, and positional dizziness) is associated with increased all‐cause and cause‐specific mortality (Lin et al. 2024). Previous meta‐analyses have shown that vertigo and gait instability were independent predictors of falls in community‐dwelling elderly individuals (Deandrea et al. 2010; Schlick et al. 2016). Falls can lead to severe consequences such as fractures and head injuries, which in turn exacerbate the physical and mental burden on patients and increasing age‐standardized mortality rates (GBD 2021 US Burden of Disease Collaborators 2024). FoF is also a major health issue poststroke, equally significant as falls themselves, and is associated with adverse outcomes such as functional decline, reduced social participation, and decreased quality of life, even increasing the risk of falls (Scarlett et al. 2019). Mild stroke accompanied by vestibular symptoms may potentially develop into a chronic and disabling condition known as persistent postural‐perceptual dizziness (PPPD) in the long term. This condition is characterized by dizziness and vertigo triggered by upright posture, self‐motion, and visual motion. Patients with PPPD often suffer a pronounced FoF (Schlick et al. 2016). Although falls and FoF are distinct geriatric syndromes, they exhibit a well‐established bidirectional relationship: A history of falls predicts subsequent FoF, while FoF independently increases fall risk and promotes fear‐avoidance behaviors that further exacerbate physical deconditioning (Asai et al. 2022; Schlick et al. 2016). Accordingly, falls and FoF were combined as a composite outcome in the present study to more comprehensively capture the risk profile of postural instability in this population.

Currently, there are few observational studies on the long‐term prognosis of patients with minor stroke accompanied by vestibular symptoms, with only a limited number of small‐sample studies available (Jiashu et al. 2022). The evolution and outcomes of vestibular symptoms in these patients have not been thoroughly explored, and the risk factors for long‐term falls/FoF in this population remain unclear. Therefore, this study aims to systematically analyze the evolution of vestibular symptoms in patients with minor stroke within 1 year, identify risk factors to predict a high risk of falls in these patients, provide references for early clinical intervention and preventive strategies, ultimately reduce the occurrence of falls/FoF, and improve the long‐term quality of life in these patients.

## 2 Methods

### 2.1 Study Population and Design

**FIGURE 1:**
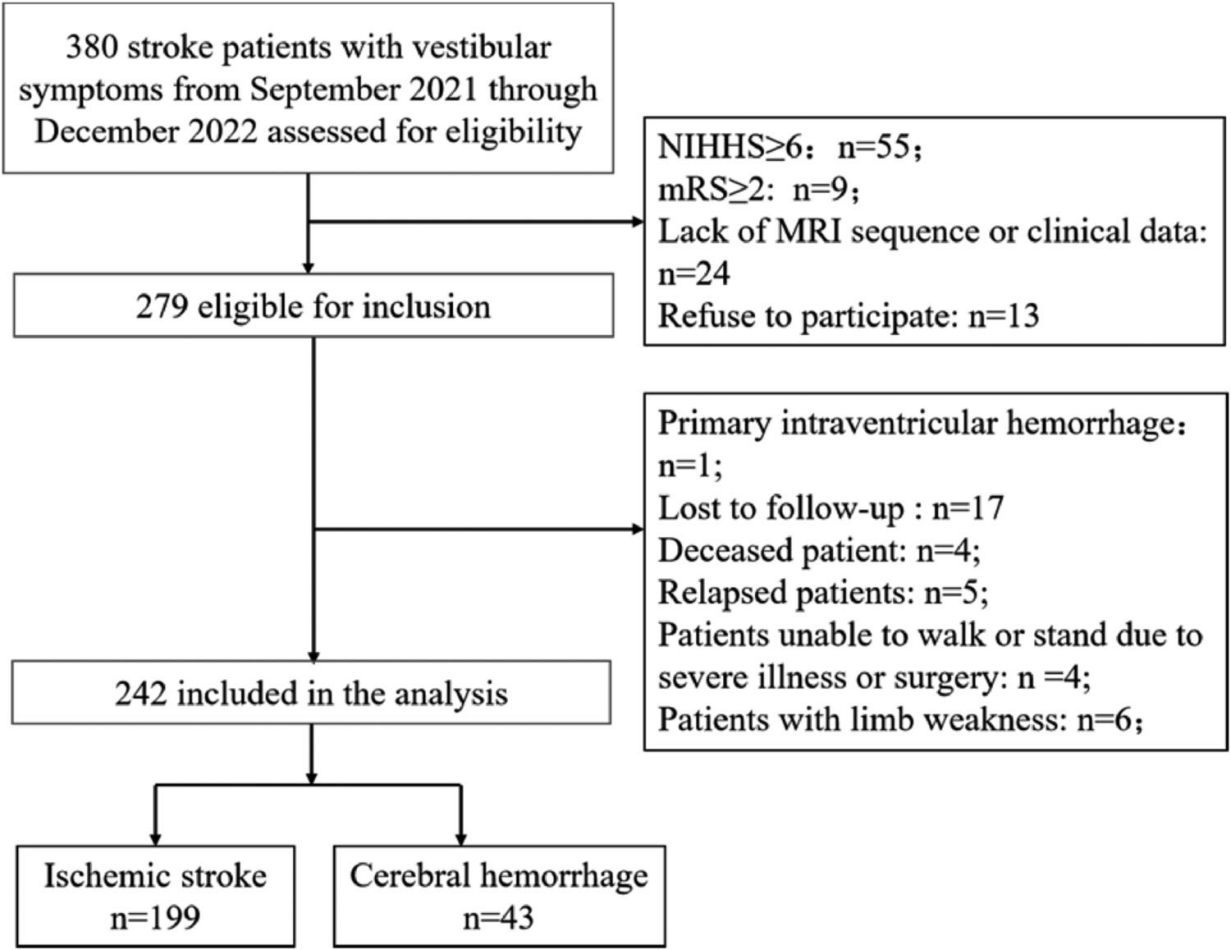
Study flow chart.

This is a prospective observational study. From September 2021 to December 2022, we consecutively recruited 380 acute minor stroke patients with vestibular symptoms (including vertigo, dizziness, vestibular‐visual symptoms, and postural symptoms) at Beijing Tiantan Hospital. The inclusion criteria were age ≥ 18 years old; meeting the diagnostic criteria for acute cerebral infarction or cerebral hemorrhage; stroke onset within 7 days; NIHSS score < 6 at admission; pre‐stroke mRS score < 2; complete medical data and available magnetic resonance imaging (MRI); and consent to participate in follow‐up. The exclusion criteria included primary intraventricular hemorrhage, subarachnoid hemorrhage, transient ischemic attack; impaired consciousness, severe aphasia, dementia, or movement disorders; absence of cranial MRI sequences; life expectancy of less than 1 year; loss to follow‐up, recurrence, or death; limb weakness due to disease or surgery at follow‐up, unable to stand or walk; and refusal to participate in this study. Based on the above criteria, a total of 242 minor stroke patients with vestibular symptoms at onset were ultimately enrolled, consisting of 199 ischemic stroke and 43 hemorrhagic stroke patients (Figure 1). This study has been approved by the Medical Ethics Committee of Beijing Tiantan Hospital, and all participants have signed written informed consent forms.

### 2.2 Clinical Information

We systematically collected demographic data (age, sex), vascular risk factors (current smoking, regular alcohol consumption, hypertension, diabetes mellitus, hyperlipidemia, and history of coronary heart disease or stroke), and relevant medical history (migraine, sleep disorders). Smoking was defined as smoking ≥ 1 cigarette daily for more than 1 year; drinking history was defined as drinking alcohol at least one time every week for more than 1 year. Vestibular symptoms were evaluated at admission through standardized bedside examinations in accordance with the Bárány Society diagnostic criteria (2009) (Bisdorff et al. 2009). Symptomatology included (1) vertigo, (2) dizziness, (3) vestibulo‐visual symptoms, and (4) postural symptoms, with symptom severity quantified using a 10‐point Visual Analog Scale (VAS) (range 0–10). Neurological impairment was evaluated using the NIH Stroke Scale (NIHSS) at admission and discharge. Bedside examination included assessment of gaze‐evoked nystagmus, as well as the finger‐to‐nose and heel‐to‐shin tests, to evaluate cerebellar and central vestibular function. Psychological status was assessed using the Hamilton Anxiety Rating Scale (HAMA) (cutoff ≥ 7 for anxiety) and Hamilton Depression Scale (HAMD) (cutoff ≥ 8 for depression).

### 2.3 Follow‐Up Assessment at 3–12 Months

A total of 242 patients underwent comprehensive 12‐month longitudinal follow‐up, with standardized assessments performed through either in‐person outpatient evaluations or structured telephone interviews at predetermined intervals: 3 months (±7 days), 6 months (±7 days), and 12 months (±14 days) post‐enrollment. At each time point, we systematically evaluated (1) functional status using the modified Rankin Scale (mRS), (2) persistence and characteristics of vestibular symptoms, and (3) psychological status employing the HAMA and HAMD rating scales. All assessments were performed by neurologists trained in neuropsychological evaluation. The 12‐month assessment specifically focused on fall‐related outcomes, incorporating three key measures: (1) self‐reported falls occurring within the preceding year, (2) degree of FoF quantified by the Activities‐specific Balance Confidence (ABC) Scale, and (3) diagnosis of PPPD based on established ICD‐11 and Bárány Society diagnostic criteria (Staab et al. 2017). The composite end point of high fall risk (encompassing both falls and FoF) was defined by meeting any of the following criteria: (1) ≥ 1 falls in the past year, (2) ABC score ≤ 67, or (3) meeting diagnostic criteria for PPPD. Patients were subsequently stratified into high‐ and low‐risk groups for subsequent analysis.

### 2.4 Imaging Assessment

Imaging data were independently analyzed by two experienced neuroradiologists using poststroke cranial CT and MRI scans to determine stroke type and location. Stroke type was classified as ischemic or hemorrhagic, with ischemic stroke further subtyped according to the TOAST criteria (Adams et al. 1993). Lesion location was first categorized by vascular territory (supratentorial stroke, infratentorial stroke, or both), then further coded using a non‐mutually‐exclusive binary strategy in which each anatomical region (cerebellum, brainstem, thalamus, basal ganglia/internal capsule, corona radiata, centrum semiovale, and frontal, parietal, temporal, and occipital lobes) was independently coded as involved or not, to accommodate the high prevalence of multifocal lesions in this population. Structural imaging assessments included the evaluation of lacunar infarcts, enlarged perivascular spaces (EPVS), cerebral microbleeds (CMBs), and white matter hyperintensities (WMH), following the STRIVE criteria (Wardlaw et al. 2013). The total SVD burden was quantified using an established four‐point scale (0–4) (Staals et al. 2014), with clinically significant burden defined as ≥ 2 points (Doubal et al. 2010). Discrepancies were resolved through consensus review with a third senior neuroradiologist.

### 2.5 Statistical Analysis

Patients were classified into two groups based on the presence or absence of high fall risk at the 1‐year follow‐up. Continuous variables with normal distribution were reported as mean ± standard deviation and compared using the *t*‐test, while non‐normally distributed variables were presented as median (Q1–Q3) and analyzed using the Mann‐Whitney *U* test. Categorical variables were expressed as frequency (%) and compared using the chi‐square test or Fisher's exact test. The Kruskal–Wallis *H* test and Dunn's test were applied for multiple comparisons of mRS scores across follow‐up time points. Variables with *p*‐value < 0.05 in univariate analysis, along with clinically relevant factors, were included in a multivariate logistic regression model. Stepwise regression was used to identify independent risk factors for high fall risk within 1 year, with odds ratios (OR) and 95% confidence intervals (CI) calculated. A *p*‐value < 0.05 was considered statistically significant. Two multivariable regression models were developed: Model 1 based on baseline characteristics and Model 2 incorporating significant variables from the 3–6 month follow‐up into Model 1. Receiver operating characteristic (ROC) curves were plotted for both models, and the areas under the curve (AUC) with 95% CI were calculated. Statistical analyses were performed using SPSS (v.26.0; IBM).

## 3 Results

### 3.1 Baseline Information and Evolution of Vestibular Symptoms

**FIGURE 2:**
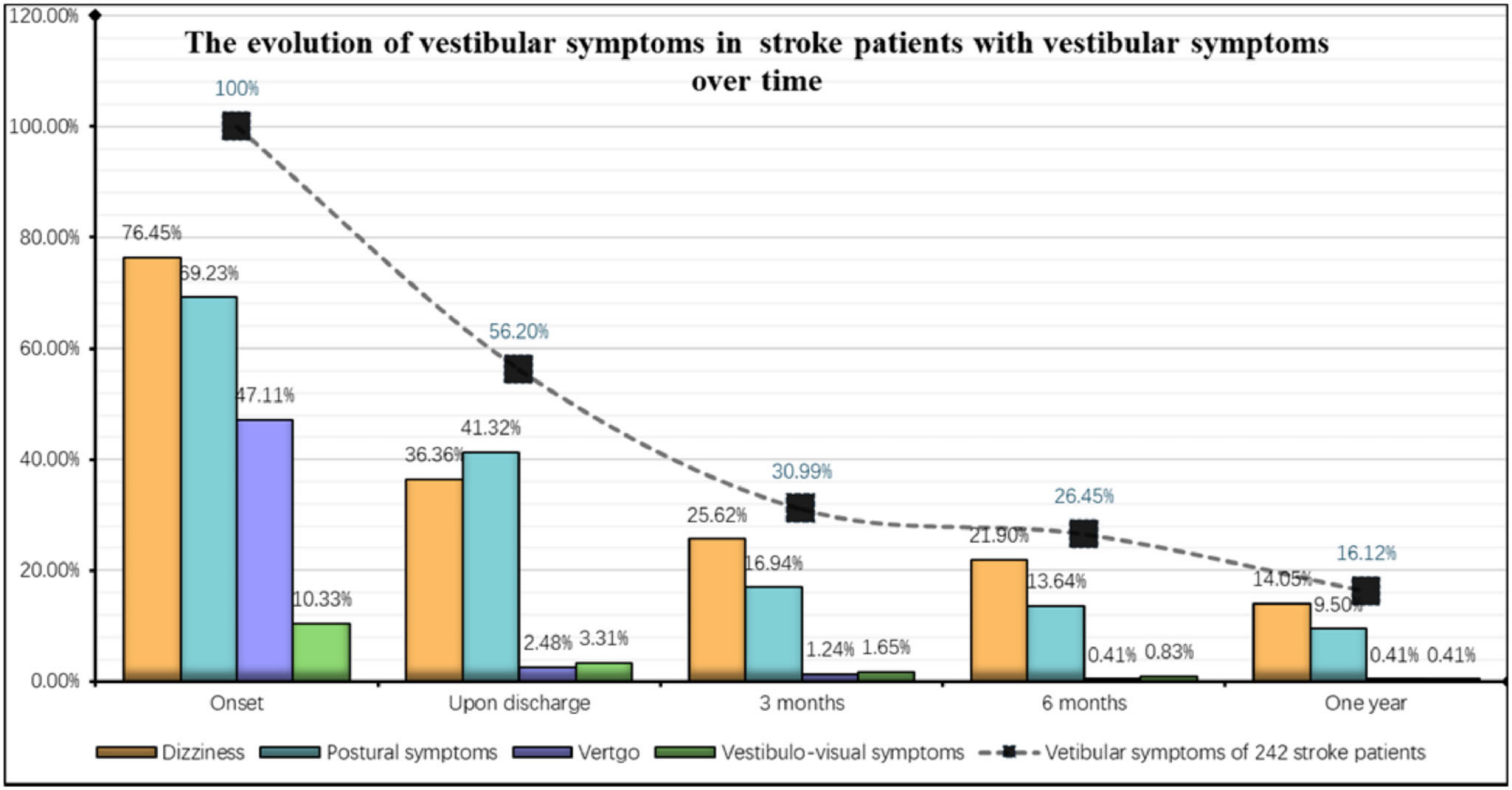
Evolution of four kinds of vestibular symptoms in 242 mild stroke patients over time.

The study consisted of 242 consecutive mild stroke patients (56.1 ± 12.3 years) who were presented with acute vestibular symptoms at onset. The longitudinal analysis revealed a distinct biphasic pattern of vestibular symptom resolution (Figure 2). At admission, dizziness (76.4%), postural symptoms (69.2%), and vertigo (47.1%) predominated, while vestibular‐visual symptoms were less common (10.3%). By discharge, 56.2% of patients remained symptomatic, primarily with dizziness (36.4%) and postural symptoms (41.3%), whereas vertigo and vestibular‐visual symptoms persisted in only 2%–3% of cases. The first 3 months poststroke demonstrated rapid symptom resolution, with prevalence declining to 31.0% (dizziness: 25.6%, postural symptoms: 16.9%). Beyond this acute phase, symptom resolution slowed considerably, with 26.4% symptomatic at 6 months and 16.1% at 12 months. Notably, the residual symptoms at 1 year were predominantly dizziness (14.1%) and postural instability (9.5%), with a subset meeting diagnostic criteria for PPPD (*n* = 20), suggesting chronic vestibular dysfunction. Despite these persistent symptoms, functional outcomes were excellent, with 97.1% achieving mRS ≤ 2 by 3 months, improving to 99.6% at 12 months. This dissociation between symptom persistence and functional recovery highlights the need for targeted vestibular rehabilitation even in patients with good overall neurological outcomes.

### 3.2 Univariate Analysis of High Fall Risk in Minor Stroke Patients With Vestibular Symptoms

This prospective study of 242 minor stroke patients with vestibular symptoms demonstrated a 16.5% incidence of falls and FoF at the 1‐year follow‐up. Baseline risk factors included advanced age, diabetes mellitus, prior stroke history, and depressive status at admission (Table 1). Higher NIHSS scores at both admission and discharge, along with more severe vestibular symptoms (VAS scores), were associated with increased fall risk. Neither gaze‐evoked nystagmus nor the heel‐knee‐shin test demonstrated a statistically significant association with falls and FoF at 12 months. A positive finger‐nose test at admission showed a nominally significant association with 12‐month falls and FoF on univariable analysis. Neuroimaging assessment revealed that, except for lesions involving the temporal lobe, which showed a significant association with fall risk (*p* < 0.05), neither stroke type nor other lesion locations (detailed lesion locations are provided in the Supporting Information) were significantly associated. Cerebral small vessel disease (CSVD) burden (SVD score ≥ 2) remained significantly associated with fall risk (*p* < 0.05). Longitudinal evaluation identified persistent vestibular symptoms—particularly dizziness and postural symptoms—as well as poorer functional status (mRS) and anxiety/depression status at 3–6 month and 12‐month follow‐ups as risk factors of 1‐year falls outcomes (Table 2). These findings underscore the multifactorial nature of poststroke fall risk, emphasizing the importance of comprehensive assessment incorporating clinical, functional, and neuroimaging markers throughout the 1st year poststroke.

**TABLE 1:** Baseline information and univariate analysis.

Variables	Total	Falls or fear of falling within 1 year after onset		*p‐*value
	*N* = 242	No, *n* = 202	Yes, *n* = 40	
Male, *n* (%)	203 (83.88)	170 (84.16)	33 (82.50)	0.794
Age, mean ± SD	56.08 ± 12.30	54.97 ± 12.24	61.70 ± 11.08	0.001
Medical history, *n* (%)				
Hypertension	160 (66.12)	131 (64.85)	29 (72.50)	0.350
Hyperlipidemia	46 (19.01)	36 (17.82)	10 (25.00)	0.290
Diabetic mellitus	72 (29.75)	54 (26.73)	18 (45.00)	0.021
Coronary heart disease	37 (15.29)	28 (13.86)	9 (22.50)	0.165
History of stroke	48 (19.83)	31 (15.35)	17 (42.50)	<0.001
History of migraine	22 (9.09)	18 (8.91)	4 (10.00)	0.827
Sleep disorder history	54 (26.73)	54 (26.73)	13 (32.50)	0.456
Smoking history	163 (67.36)	134 (66.34)	29 (72.50)	0.448
Drinking history	151 (62.40)	127 (62.87)	24 (60.00)	0.732
Type of stroke, *n* (%)				0.961
Ischemic stroke	199 (82.23)	166 (82.18)	33 (82.50)	
Cerebral hemorrhage	43 (17.77)	36 (17.82)	7 (17.50)	
Toast classification in ischemic stroke				0.676
Large‐artery atherosclerosis	175 (87.94)	143 (86.14)	32 (96.97)	
Cardioembolism	7 (3.52)	7 (4.22)	0 (0.00)	
Small‐vessel occlusion	1 (0.50)	1 (0.60)	0 (0.00)	
Stroke of other determined etiology	9 (4.52)	8 (4.82)	1 (3.03)	
Stroke of undetermined etiology	7 (3.52)	7 (4.22)	0 (0.00)	
Location of stroke, *n* (%)				
Supratentorial stroke	64 (26.45)	53 (26.2)	11 (27.50)	0.869
Infratentorial stroke	149 (61.57)	126 (62.40)	23 (57.50)	0.562
Supratentorial + infratentorial stroke	29 (11.98)	23 (11.40)	6 (15.00)	0.520
Temporal lobe involvement	19 (7.85)	12 (5.94)	7 (17.50)	0.022
SVD‐scores ≥ 2, *n* (%)	107 (44.21)	79 (39.11)	28 (70.00)	<0.001
Clinical characteristics, *n* (%)				
NIHSS score at onset, median (Q1, Q4)	2 (1, 3)	2 (0, 3)	2 (1, 4)	0.036
NIHHS score at discharge, median (Q1, Q4)	1 (0, 3)	1 (0, 2)	2 (1, 3.8)	0.003
Type of vestibular symptoms, *n* (%)				
Dizziness	185 (76.45)	154 (76.24)	31 (77.50)	0.864
Vertigo	114 (47.11)	93 (46.04)	21 (52.50)	0.455
Postural symptoms	162 (69.23)	131 (67.53)	31 (77.50)	0.213
Vestibulo‐visual symptoms	25 (10.33)	20 (9.90)	5 (12.50)	0.622
VAS scores (vestibular symptoms), median (Q1, Q4)	8 (5, 10)	7(5, 10)	8.5 (7, 10)	0.002
Gaze‐evoked nystagmus	73 (30.17%)	60 (29.70%)	13 (32.50%)	0.870
Direction of nystagmus				0.261
Horizontal nystagmus	70 (28.93%)	58 (28.71%)	12 (30.00%)	
Torsional nystagmus	2 (0.83%)	2 (0.99%)	0 (0.00%)	
Vertical nystagmus	1 (0.41%)	0 (0.00%)	1 (2.50%)	
Heel‐to‐shin test				0.060
Normal	142 (58.7%)	125 (61.9%)	17 (42.5%)	
Abnormal	79 (32.6%)	60 (29.7%)	19 (47.5%)	
Unable to assess	21 (8.7%)	17 (8.4%)	4 (10.0%)	
Finger‐to‐nose test				0.025
Normal	138 (57.0%)	122 (60.4%)	16 (40.0%)	
Abnormal	87 (36.0%)	65 (32.2%)	22 (55.0%)	
Unable to assess	17 (7.0%)	15 (7.4%)	2 (5.0%)	
Anxiety, *n* (%)	39 (16.12)	29 (14.36)	10 (25.00)	0.094
Depression, *n* (%)	39 (16.12)	28 (13.86)	11 (27.50)	0.032

**TABLE 2:** Longitudinal follow‐up evaluation information.

Variables	Total	Falls or fear of falling		*p*‐value
		No	Yes	
3‐month follow‐up				
mRS score, median (Q1, Q4)	1 (0, 1)	1 (0, 1)	1 (1, 2)	<0.001
Vestibular symptom, *n* (%)	75 (30.99)	46 (22.77)	29 (72.50)	< 0.001
Dizziness	62 (25.62)	38 (18.81)	24 (60.00)	<0.001
Vertigo	3 (1.24)	2 (0.99)	1 (2.50)	0.430
Vestibulo‐visual symptom	4 (1.65)	3 (1.49)	1 (2.50)	0.646
Postural symptom	41 (16.94)	26 (12.87)	15 (37.50)	<0.001
Anxiety and/or depression, *n* (%)	37 (15.29)	21 (10.40)	16 (40.00)	<0.001
6‐month follow‐up				
mRS score, median (Q1, Q4)	1 (0, 1)	1 (0, 1)	1 (0, 2)	0.002
Vestibular symptom, *n* (%)	64 (26.45)	36 (17.82)	28 (70.00)	<0.001
Dizziness	53 (21.90)	30 (14.85)	23 (57.50)	<0.001
Vertigo	1 (0.41)	1 (0.50)	0 (0.00)	0.656
Vestibulo‐visual symptom	2 (0.83)	1 (0.50)	1 (2.50)	0.201
Postural symptom	33 (13.64)	18 (8.91)	15 (37.50)	<0.001
Anxiety and/or depression	28 (11.57)	16 (7.92)	12 (30.00)	<0.001
1‐year follow‐up				
mRS score, median (Q1, Q4)	0 (0, 1)	0 (0, 1)	1 (0, 1.8)	<0.001
Vestibular symptom, *n* (%)	39 (16.12)	15 (7.43)	24 (60.00)	<0.001
Dizziness	34 (14.05)	14 (6.93)	20 (50.00)	<0.001
Vertigo	1 (0.41)	1 (0.50)	0 (0.00)	0.656
Vestibulo‐visual symptom	1 (0.41)	0 (0.00)	1 (2.50)	0.024
Postural symptom	23 (9.50)	5 (2.48)	18 (45.00)	<0.001
Anxiety and/or depression, *n* (%)	27 (11.16)	14 (6.93)	13 (32.50)	<0.001

### 3.3 Multivariate Logistic Regression Analysis

**FIGURE 3:**
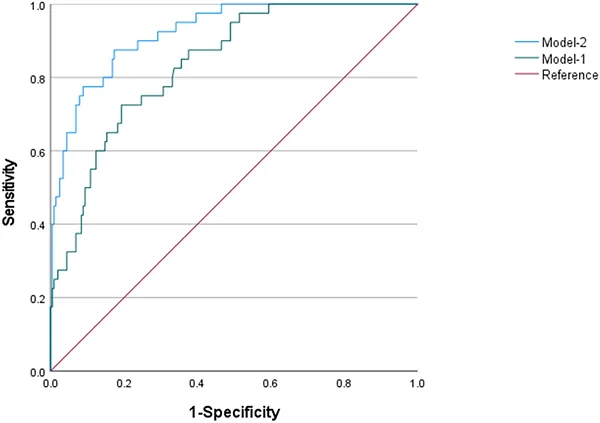
The ROC curve for predicting falls and fear of falling within 1 year in mild stroke patients with vestibular symptoms.

To identify independent risk factors for 1‐year fall risk, we developed two binary logistic regression models based on univariate analysis and clinical relevance. As shown in Table 3, Model 1 incorporated significant baseline characteristics from Table 1, with stepwise forward selection identifying six independent predictors: advanced age, prior stroke history, severe vestibular symptoms at admission, depression, temporal lobe (OR = 5.694, 95% CI: 1.517–21.374), SVD score ≥ 2 (OR = 2.917, 95% CI: 1.212–7.016), and higher NIHSS scores, achieving good discrimination (AUC: 0.836, 95% CI: 0.776–0.897). Based on Model 1, Model 2 additionally incorporated significant 3–6 month follow‐up variables, demonstrating that 3‐month anxiety/depression (OR = 5.583, 95% CI: 1.756–17.749) and persistent vestibular symptoms at 6 months (OR = 14.281, 95% CI: 4.815–42.355) further improved predictive accuracy (AUC: 0.914, 95% CI: 0.866–0.963) (Figure 3). These models provide clinically useful tools for fall risk stratification in minor stroke patients with vestibular symptoms.

**TABLE 3:** Multiple logistic regression analysis.

Predictor	Model 1			Model 2		
Baseline data	*p*‐value	OR	95% CI	*p*‐value	OR	95% CI
Age	0.037	1.043	1.002–1.085	0.020	1.057	1.009–1.107
History of stroke	0.020	2.848	1.180–6.870	0.021	3.522	1.214–10.219
VAS score of vestibular symptoms at onset	0.001	1.443	1.157–1.799	0.008	1.435	1.099–1.872
NIHSS score at discharge	0.002	1.533	1.175–2.001	0.001	1.776	1.263–2.498
Depression	0.009	3.625	1.387–9.476	—	—	—
SVD scores ≥ 2	0.017	2.917	1.212–7.016	0.002	6.166	1.985–19.153
Temporal lobe involvement	0.010	5.694	1.517–21.374	0.021	7.005	1.346–36.463

3‐month follow‐up						
Anxiety and/or depression				0.004	5.583	1.756–17.749

6‐month follow‐up						
Vestibular symptom				<0.001	14.281	4.815–42.355
AUC value	0.836(0.776–0.897)			0.923(0.882–0.963)		

*Note*: Model 1**—** Adjusted age, diabetes mellitus, stroke story, NIHSS score at admission, NIHSS score at discharge, baseline vestibular symptom severity (VAS score), depression at admission, total SVD score ≥ 2, temporal lobe involvement, and finger‐to‐nose test. Model 2**—**Adjusted age, diabetes mellitus, prior stroke story, NIHSS score at admission, NIHSS score at discharge, baseline vestibular symptom severity (VAS score), depression at admission, total SVD score ≥ 2, temporal lobe involvement, finger‐to‐nose test, vestibular symptoms at 3/6 month, anxiety and/or depression at 3/6 month, and mRS score at 3/6 month.

## 4 Discussion

This study describes the longitudinal characterization of vestibular symptom resolution in minor stroke patients within a 12‐month period and identifies independent predictors of fall risk in this specific population. The findings demonstrated that advanced age, prior stroke history, higher vestibular symptom severity (VAS score), NIHSS score, and a total SVD score ≥ 2 at baseline are independent predictors of high fall risk within 1 year. Furthermore, during follow‐up, the presence of anxiety and/or depression at 3 months and persistent vestibular symptoms at 6 months emerged as significant predictors of falls or FoF.

### 4.1 Biphasic Resolution of Poststroke Vestibular Symptoms

The study is the first longitudinal investigation into the spatiotemporal evolution of vestibular symptoms within a 12‐month period poststroke, revealing the temporal pattern of vestibular symptom recovery: rapid early improvement followed by gradual late resolution. Dizziness (76.45%) and postural symptom (69.23%) are the most common manifestations. Studies suggest that early symptom resolution may result from spontaneous vestibular system recovery, a well‐documented form of post‐lesion neural plasticity (Lacour 2006). Additionally, “top‐down” vestibular compensation—mediated by the vestibular nuclei, vestibular cortex, and cerebellum—facilitates adaptation, sensory substitution, and habituation, though recovery varies among individuals (Lacour et al. 2016). At 6‐month follow‐up, 26.45% (64/242) of patients demonstrated persistent vestibular symptoms, with 16.12% (39/242) remaining symptomatic at 12 months. This persistent symptomatology may reflect either irreversible neuronal injury or failure of vestibular compensation mechanisms. Notably, these patients frequently developed comorbid neuropsychiatric conditions, particularly anxiety and depression, which may contribute to the progression toward PPPD. Patients with PPPD persisting over a year exhibit more severe anxiety and functional impairment than those with shorter disease duration (Teh and Prepageran 2022; Xing et al. 2024). This study incorporated PPPD diagnosis into the 1‐year follow‐up as a composite outcome to assess its long‐term impact. Furthermore, the study observed continuous neurological improvement in mild stroke with vestibular symptoms, with mRS scores declining over time. A long‐term follow‐up of patients with vestibular stroke (acute brainstem‐cerebellar stroke with vestibular symptoms) similarly reported a significant decrease in mRS scores (from 2.1 to 1.1, *p* < 0.001) (Schuhbeck et al. 2022). While mRS and NIHSS are widely used for evaluating mild stroke, the floor effect of the mRS score limits its sensitivity in detecting vestibular and cognitive‐affective impairments. A previous cohort study found that patients with dizziness had significantly higher all‐cause mortality (45.6% vs. 27.1%) and increased mortality from diabetes, cardiovascular diseases, and cancer (Lin et al. 2024). By tracking poststroke vestibular symptoms and fall risk over an extended period, this study addresses the end point limitations of prior stroke research and provides a more comprehensive prognostic evaluation.

### 4.2 The Relationship Between Vestibular Symptoms and Fall Risk

The 2022 World Falls Guidelines highlight dizziness as a modifiable risk factor for falls, with this study extending these findings to demonstrate its longitudinal impact (Montero‐Odasso et al. 2022). This study found that the severity of vestibular symptoms at onset (VAS score: OR = 1.410; 95% CI: 1.090–1.824) and persistent vestibular symptoms at 6 months (OR = 12.754; 95% CI: 4.555–35.712; *p* < 0.001) were independent predictors of elevated fall risk at 1 year. A recent meta‐analysis of over 100,000 participants across 14 studies corroborates these results, showing that dizziness significantly increased fall risk over 6 months to 11 years (OR = 1.63; 95% CI: 1.44–1.84; *p* < 0.001) (Li et al. 2024). However, these studies primarily involved community‐dwelling older adults, with benign paroxysmal positional vertigo (BPPV) and other peripheral vestibular disorders, limiting generalizability to other populations. This study extends these findings by demonstrating that ​central vestibular impairment, characterized by milder but prolonged symptoms, may confer a greater long‐term risk of falls and FoF compared to peripheral dysfunction. Larger studies are needed to confirm these findings. Notably, VAS scores for vestibular symptoms may serve as an indirect measure of central vestibular system damage and its associated fiber pathways, offering potential predictive value for fall risk and FoF within 1 year poststroke.

### 4.3 The Relationship Between Temporal Lobe Involvement and Fall Risk

Temporal lobe involvement was independently associated with 12‐month fall risk in our cohort. The temporal and insular cortices including the parieto‐insular vestibular cortex (PIVC) serve as the primary cortical hub for vestibular signal processing and postural compensation (Dieterich and Brandt 2015; zu Eulenburg et al. 2012). While brainstem and cerebellar lesions dominate the acute clinical presentation, the integrity of temporal‐insular vestibular cortical networks becomes increasingly critical as spontaneous compensation progresses. We therefore propose that temporal lobe lesions impair cortical re‐weighting and adaptive compensation mechanisms, thereby elevating long‐term fall risk. This is consistent with evidence linking insular and temporal cortex lesions to impaired vestibular compensation and prolonged postural instability in chronic stroke (Baier et al. 2021; Karnath et al. 2000).

### 4.4 The Relationship Between Total SVD Score and Fall Risk in Poststroke Vestibular Disorders

This study illustrated that total SVD score ≥ 2 is an independent risk factor of falls or FoF within 1 year in minor stroke patients with vestibular symptoms (*p* = 0.003; OR = 5.106; 95% CI: 1.747–14.921). A systematic review and meta‐analysis of 73 studies on gait, balance, and falls in CSVD revealed significant associations between WMH burden, lacunar infarct count, and fall risk across both prospective and retrospective cohorts (Sharma et al. 2023). The total SVD score offers a simple yet comprehensive assessment of the overall impact of SVD on the brain, but its direct relationship with falls remains unexplored in cross‐sectional or longitudinal studies (Staals et al. 2014). A prior study of 461 patients with lacunar and cortical infarcts reported an approximately 37% prevalence of SVD scores ≥ 2, while this study identified 44.21% of patients with vestibular symptoms at onset had SVD scores ≥ 2 (Staals et al. 2014). SVD lesions may contribute to fall risk by directly disrupting the frontal cortical‐subcortical network or indirectly impairing cognition‐related structures, leading to executive dysfunction. Further research is warranted to investigate how CSVD‐related brain network alterations affect stroke patients with vestibular symptoms (Parihar et al. 2013).

### 4.5 Anxiety, Depression, and Fall Risk in Patients With Minor Stroke Accompanied by Vestibular Symptoms

This study found that patients with anxiety and/or depression at the 3‐month follow‐up had a significantly higher risk of falls or FoF at 1 year. Anxiety and depression are closely linked to secondary functional dizziness in vestibular disorders and negatively impact stroke recovery (Chun et al. 2018; Cousins et al. 2017). Prior research has shown that increased depressive and anxiety symptoms significantly contribute to the development and persistence of FoF (RR = 1.36, 95% CI: 1.16–1.60) (Choi et al. 2022). While anxiety alone has not been identified as a direct fall risk factor in large prospective studies, FoF—strongly associated with anxiety—can impair balance and gait and independently predict recurrent falls in elders (Delbaere et al. 2010). This finding supports the composite outcome design of this study. Additionally, depression is linked to frailty, social isolation, reduced daily activity, and poorer physical function, all of which may increase fall risk. These results underscore the importance of long‐term emotional and psychological monitoring in stroke patients, particularly those with vestibular symptoms, even if their initial gait and balance function appears intact poststroke.

### 4.6 Limitations

Despite its clinical significance in the long‐term follow‐up of minor stroke patients with vestibular symptoms, this study has certain limitations. First, the observed incidence of falls and FoF (16.5%) was lower than the pooled global prevalence (∼50%) reported in systematic reviews, likely reflecting both the retrospective ascertainment of falls at the 12‐month follow‐up—a method prone to underestimation, though 12‐month recall has demonstrated acceptable sensitivity and specificity in stroke populations (Cummings et al. 1988; Kunkel et al. 2011)—and the comparatively preserved functional status of this understudied minor stroke population. Second, patients with vestibular anosognosia may be at even higher risk due to impaired awareness of balance deficits, representing a subgroup warranting dedicated investigation (Hadi et al. 2025). Third, the ABC Scale was selected over the Falls Efficacy Scale‐International (FES‐I) given its established validity in stroke and vestibular dysfunction patients and its formal recommendation by the Neurology Section of the American Physical Therapy Association (Alghwiri et al. 2016; Kobayashi et al. 2025); future studies incorporating both instruments would facilitate cross‐validation across populations. Fourth, although bedside oculomotor and cerebellar signs were documented and analyzed, objective bithermal caloric test were contraindicated in the acute stroke setting; future prospective studies should incorporate objective vestibular and balance assessments at baseline to better characterize the vestibular‐balance profile at stroke onset and allow more rigorous covariate adjustment. Fifth, rehabilitation interventions were not systematically recorded during follow‐up, which limits our ability to disentangle the independent contributions of therapeutic intervention and spontaneous neurological recovery to the observed outcomes. This confounding effect may be partially mitigated by the single‐center design and consistent institutional management protocols, though future studies should systematically document rehabilitation exposure as a covariate.

## 5 Conclusion

In mild stroke patients with vestibular symptoms, symptom resolution is markedly accelerated within the initial 3‐month period, followed by a deceleration in recovery trajectory. Early fall risk can be predicted by the severity of vestibular symptoms and CSVD burden. Regular follow‐ups assessing persistent vestibular symptoms and emotional states are crucial for early identification of high‐risk patients. Our study suggests three critical intervention windows for fall prevention: (1) acute phase (0–3 months) for vestibular symptom management, (2) subacute phase (3–6 months) for psychological comorbidity screening, and (3) chronic phase (6–12 months) for targeted fall prevention strategies. Particular attention should be given to patients with SVD burden, severe initial symptoms, psychological comorbidities, or temporal lobe involvement who may benefit most from early implementation of combined vestibular rehabilitation and cognitive‐behavioral interventions. This stratified approach may help reduce long‐term fall risk in this vulnerable patient population.

## Author Contributions

**Jiaokun Jia**: investigation, project administration. **Jiashu Li**: investigation, project administration. **Yao Zhong**: writing – original draft, visualization, investigation. **Ruixuan Jiang**: investigation, project administration. **Xingquan Zhao**: conceptualization, resources, writing – review and editing. **Yi Ju**: conceptualization, writing – review and editing, resources.

## Funding

This research was supported by the Clinical and Translational Medicine Research Program from Chinese Academy of Medical Sciences (2022‐I2M‐C&T‐B‐116), the Science Innovation Brain Science and Brain‐like Research from the Ministry of National Science and Technology, China (2022ZD0211805), and the National Key Research and Development Program of China (2022YFC3501102).

## Ethics Statement

This study involves human participants and was approved by the Ethics Committee in Beijing Tiantan Hospital (KY 2021‐055‐02). Participants gave informed consent to participate in the study before taking part.

## Conflicts of Interest

The authors declare no conflicts of interest.

## Supporting information




**Supplementary Material**: brb371509‐sup‐0001‐SuppMat.docx

## Data Availability

The data and datasets used or analyzed during the current study are available from the corresponding author on reasonable request.
