# Supplementary material for: Falls and Fear of Falling in Minor Stroke Patients With Vestibular Symptoms: A Longitudinal Observational Study
Source: Brain Behav. 2026 Jun 3;16(6):e71509. doi: 10.1002/brb3.71509 (PMC13580758; doi:10.1002/brb3.71509)
Supplement: Supplementary file 1 [file BRB3-16-e71509-s001.docx]

**Supplements Table 1. Detailed Characteristics of Lesion Location and Stroke Type**

| **Variables** | **Total** | **Falls or fear of falling** | | ***P* value** |
| --- | --- | --- | --- | --- |
|  |  | **No** | **Yes** |  |
|  | N=242 | No, n=202 | Yes, n=40 |  |
| Type of stroke |  |  |  | 0.961 |
| Hemorrhage | 43 (17.77%) | 36 (17.82%) | 7 (17.50%) |  |
| Ischemic stroke | 199 (82.23%) | 166 (82.18%) | 33 (82.50%) |  |
| TOAST classification in ischemic stroke |  |  |  | 0.676 |
| Large-artery atherosclerosis | 175 (87.94%) | 143 (86.14%) | 32 (96.97%) |  |
| Cardioembolism | 7 (3.52%) | 7 (4.22%) | 0 (0.00%) |  |
| Small-vessel occlusion | 1 (0.50%) | 1 (0.60%) | 0 (0.00%) |  |
| Stroke of other determined etiology | 9 (4.52%) | 8 (4.82%) | 1 (3.03%) |  |
| Stroke of undetermined etiology | 7 (3.52%) | 7 (4.22%) | 0 (0.00%) |  |
| Lesion location |  |  |  |  |
| Cerebellum | 100 (41.32%) | 83 (41.09%) | 17 (42.50%) | 0.869 |
| Brainstem | 103 (42.56%) | 87 (43.07%) | 16 (40.00%) | 0.854 |
| Pons | 64 (26.45%) | 54 (26.73%) | 10 (25.00%) | 0.975 |
| Medulla oblongata | 38 (15.70%) | 31 (15.35%) | 7 (17.50%) | 0.917 |
| Midbrain | 4 (1.65%) | 4 (1.98%) | 0 (0.00%) | 0.369 |
| Thalamus | 29 (11.98%) | 24 (11.88%) | 5 (12.50%) | 0.912 |
| Parietal lobe | 22 (9.09%) | 16 (7.92%) | 6 (15.00%) | 0.222 |
| Temporal lobe | 19 (7.85%) | 12 (5.94%) | 7 (17.50%) | 0.022 |
| Frontal lobe | 16 (6.61%) | 12 (5.94%) | 4 (10.00%) | 0.311 |
| Occipital lobe | 35 (14.46%) | 26 (12.87%) | 9 (22.50%) | 0.182 |
| Corona radiata / centrum semiovale | 18 (7.44%) | 17 (8.42%) | 1 (2.50%) | 0.322 |
| Basal ganglia / internal capsule | 25 (10.33%) | 23 (11.39%) | 2 (5.00%) | 0.391 |
